# Self-Assembled Peptides: A New Generation of Vaccine Adjuvant Platform

**DOI:** 10.3390/vaccines13121183

**Published:** 2025-11-21

**Authors:** Miao-Miao Zhang, Ji Zhu, Zhao-Yi Wang, Yu-Lun Bai, Hai-Bo Li, Buhe Nashun, Yue Jiang

**Affiliations:** 1 Inner Mongolia Key Laboratory for Molecular Regulation of the Cell, School of Life Sciences, Inner Mongolia University, Hohhot 010070, China; zmm15249454345@163.com; 2 National Engineering Research Center of Immunological Products, Department of Microbiology and Biochemical Pharmacy, College of Pharmacy, Third Military Medical University (Army Medical University), Chongqing 400038, China; zhuji0409@163.com (J.Z.); 15707231319@163.com (Z.-Y.W.); baiyulun0219@163.com (Y.-L.B.)

**Keywords:** self-assembly peptides, vaccines, nanostructures, adjuvants

## Abstract

The advent of precision medicine has spotlighted subunit and peptide-based vaccines, which offer high safety but often require potent adjuvants to enhance immunogenicity. Self-assembled peptides have emerged as a promising adjuvant platform due to their ease of synthesis, excellent biocompatibility, and tunable structural properties. Recent advances highlight their potential in boosting vaccine efficacy, with self-assembled peptides forming highly ordered architectures that are conducive to immune system activation. This review discusses the key factors driving peptide self-assembly and explores their evolving role as innovative vaccine adjuvants, alongside challenges and future development directions.

## 1. Introduction

Vaccines are among the most cost-effective public health interventions for preventing infectious diseases [1]. Successful vaccination relies on a strong and long-lasting protective immune response induced by the antigen, and adjuvants play a crucial role in this process, especially for antigens with weak immunogenicity such as high-purity recombinant proteins and synthetic peptides [2,3]. The term “adjuvant” derives from the Latin elements “*ad-*” and “*-juvare*”, which together mean “to help”. In immunology, adjuvants are non-specific immune modulators that enhance the immunogenicity and efficacy of vaccine antigens when administered alongside or prior to vaccination. They also help steer the immune response and reduce the necessary antigen dose [4,5,6,7]. However, conventional adjuvants have several limitations. For instance, aluminum-based adjuvants are linked to excessive inflammatory reactions and an inability to stimulate cellular immune responses [8,9]; oil emulsion adjuvants are difficult to inject and often cause local redness, swelling, and pain [10]; liposomal adjuvants suffer from poor stability and inconsistent encapsulation efficiency [11]; and immunostimulatory adjuvants may cause dose-dependent hemolysis and exhibit significant batch-to-batch variability [12]. As a result, there is an urgent need to develop safer, more effective adjuvants that can promote a stronger Th1-type cellular immune response.

Amino acids, the fundamental building blocks of life, can be linked in various sequences to form peptides and proteins with intricate structures and diverse biological functions [13]. Under specific physicochemical conditions, peptides can undergo spontaneous or stimulus-induced organization into defined structures or aggregates via non-covalent interactions, forming self-assembling peptides (SAPs). These interactions—including hydrogen bonding, van der Waals forces, electrostatic attractions, and hydrophobic effects—facilitate the formation of ordered nanostructures with distinct morphologies and functionalities [14,15]. Since the early 1990s, studies in the field of self-assembled peptides have increased significantly. Zhang et al. unexpectedly designed and synthesized the first self-assembled peptide EAK16 from yeast protein. This peptide can be self-assembled into nanofibers, which then form a stable hydrogel through complementary ionic interactions [16,17]. Subsequently, Later, the RADA16-I and RADA16-II peptides were engineered by replacing lysine and glutamic acid residues with arginine and aspartic acid, respectively. These peptides formed nanofibers approximately 10 nm in diameter, which entangled to create highly hydrated scaffold-like hydrogels in aqueous environments [18,19]. In another seminal study, Reches et al. demonstrated that diphenylalanine (FF) dipeptides possess remarkable self-assembly capabilities, giving rise to a range of nanostructures—including nanotubes, nanoribbons, nanofibers, and vesicles—each with distinct morphological and functional attributes [20]. Building upon the strategy of incorporating hydrophobic motifs to promote self-assembly, the Collier lab developed the Q11 peptide, which spontaneously forms β-sheet-rich nanofibers. Importantly, the Q11 system has been extensively validated for its capacity to co-assemble with antigens and facilitate their delivery to antigen-presenting cells (APCs), thereby enhancing immunogenicity [21,22,23,24]. Owing to their exceptional biocompatibility, biodegradability, low immunogenicity, and the ability to be precisely engineered at the molecular level, SAPs have emerged as a promising platform in biomedical applications. These properties have positioned SAPs as a rapidly advancing frontier in materials science and biomedicine [23,24,25,26,27,28].

Previous studies have shown that peptide-based biomaterials can be used to modulate the immune system and subsequently regulate immune responses [29,30]. Among these, SAPs are particularly advantageous due to their capacity for the modular and site-specific incorporation of multiple functional components. Through the strategic conjugation of epitopes, antigens, and immunomodulatory moieties onto the SAP backbone, it is possible to construct vaccine platforms with enhanced immunogenicity, prolonged stability, intrinsic adjuvant properties, and sustained antigen presentation [31,32,33]. Beyond their application as immune-stimulating agents in vaccine development, SAPs also show significant potential as delivery vehicles for immune factors [34]. Their high biocompatibility, multivalent presentation capabilities, and structural adaptability enable them to co-assemble immunogenic peptide epitopes with inert carrier sequences, thereby improving antigen delivery while minimizing off-target immune activation and reducing adverse side effects [35].

In recent years, SAPs have garnered significant attention as a promising platform for vaccine adjuvants. These peptides not only demonstrate an impressive ability to effectively deliver antigens, but their intrinsic self-assembly properties often result in self-adjuvant effects [36]. This review begins by providing a comprehensive overview of the key concepts, classification, and historical evolution of vaccine adjuvants. We then proceed to a detailed examination of the structural characteristics and physicochemical properties of SAPs. In addition, we highlight the distinct advantages and applications of SAPs as adjuvants in vaccine formulations, offering valuable insights into their potential for future design and development.

## 2. Vaccine Adjuvants: From Empirical Approaches to Rational Design

### 2.1. The Core Mechanism of Action of Adjuvants

Studies have shown that most adjuvants do not directly stimulate lymphocytes but act as pathogen-associated molecular patterns or damage-associated molecular patterns (DAMPs) to bind with key components of the innate immune system, thereby activating pattern recognition receptors (PRRs) and their downstream signaling pathways. This further reshapes the function of antigen-presenting cells and creates a microenvironment conducive to initiating adaptive immune responses [37,38]. The mechanisms of action of adjuvants mainly include forming antigen depots, recruiting immune cells, activating inflammasomes, enhancing MHC molecule-mediated antigen presentation, and regulating immune responses [39]. Traditionally, adjuvants have been employed to enhance the magnitude of vaccine-induced adaptive immune responses, with their evaluation typically based on antibody titers or protective efficacy against infection. However, in recent years, the role of adjuvants has expanded beyond merely enhancing the magnitude of immune responses to also directing the type of adaptive immune response, thereby enabling the induction of the most effective immune protection against specific pathogens. This area is gaining increasing attention [40].

### 2.2. Classification and Development History of Traditional Vaccine Adjuvants

Vaccine adjuvants can be classified into delivery system adjuvants, immunostimulants, and composite adjuvants based on their mechanisms of action [41]. Delivery system adjuvants primarily elicit broader and more durable immune responses by modulating the timing and magnitude of antigen delivery to APCs. Examples include aluminum-based adjuvants, oil-in-water emulsions such as MF59 and AS03, and microparticle-based systems such as virus-like particles, nanoparticles, and liposomes. In contrast, immunostimulatory agents enhance the maturation and activation of APCs through specific engagement of receptors on these cells; representative examples include Toll-like receptor (TLRs) agonists, cytokines, polysaccharides, and chitosan [1]. A detailed classification of selected adjuvants is presented in Table 1. Since the discovery of aluminum adjuvants, vaccine adjuvants have gone through a century of development. In 1926, Alexander Glenny first demonstrated that aluminum salts could significantly enhance the immune response to diphtheria toxoid. Since then, aluminum salts—recognized as the earliest adjuvants approved for human use—have been widely utilized in vaccine formulations [42,43]. From 1930 to 1947, Freund developed Freund’s adjuvant, which was capable of inducing a strong immune response; however, it was not approved for human use due to its high toxicity [38]. It was not until 1997 that the oil-in-water emulsion MF59 was approved in Europe as an adjuvant for seasonal influenza vaccines, marking the first non-aluminum adjuvant to be authorized for human use. Over the following two decades or so, based on a deep understanding of the mechanisms of innate immunity, various novel adjuvants such as AS04, AS03, AS01, Matrix-M™ and CpG ODN 1018 were successively approved for human vaccines [44], marking a new stage of rational design in adjuvant research and development.

### 2.3. Current Limitations of Existing Adjuvants and the Need for Developing Novel Adjuvants

Although the currently approved adjuvants for human use have made significant contributions to vaccine development, there are still certain limitations. Conventional adjuvants are commonly associated with both local and systemic adverse reactions. Although their primary mode of action involves inducing humoral immunity to confer protection, they are generally inadequate in eliciting strong and long-term T-cell immune responses. Additionally, a considerable proportion of conventional adjuvants fail to produce sufficient serum conversion rates or reach the protective antibody titers necessary for effective immune defense [45,46]. Furthermore, vaccine responsiveness in healthy adults tends to diminish progressively after the age of 40 to 50 [47]. Therefore, the existing limitations highlight the continued need to develop adjuvants with well-defined mechanisms, proven safety and efficacy, and rational design. This will enhance both protective antibody and cellular immune responses, thereby inducing broader immune protection, particularly in populations with suboptimal responses to current vaccines [37]. SAPs have emerged as a promising strategy in this context.

## 3. The Basic Theory of SAPs

**Figure 1:**
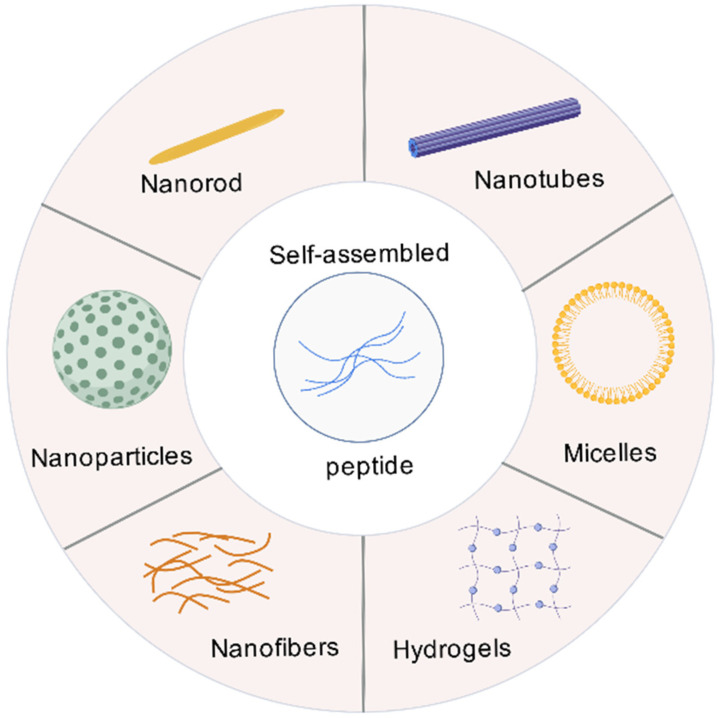
**Self-assembled structures of peptides.** SAPs form secondary structures via non-covalent interactions, which then aggregate into nanostructures, including nanorods, nanotubes, nanoparticles, nanofibers, hydrogels, and micelles.

SAPs are a class of biomaterial building blocks with unique molecular recognition capabilities. They are typically composed of amphiphilic sequences or alternately arranged amino acid sequences with positive and negative charges. Molecular self-assembly arises from dynamic equilibrium and is the process by which basic molecular components organize and coordinate effectively to form ordered supramolecular nanostructures or microstructures, such as nanofibers, nanotubes, nanoribbons and hydrogels, through non-covalent interactions under defined conditions [48,49,50] (Figure 1).

The controllability and reproducibility of the self-assembly process are due to its responsiveness to internal and external factors. Internal factors primarily refer to the intrinsic properties of the peptide molecule. These include the balance between hydrophilic and hydrophobic properties, which is dictated by the amino acid sequence; charge distribution; capacity to form hydrogen bonds; molecular weight; and propensity to adopt specific secondary structures, such as natural motifs like β-sheets, β-turns, and α-helices [51,52,53]. These intrinsic properties allow peptides to be designed in a rational way, using specific secondary structural motifs to direct the formation of complex nanostructures [54,55,56]. External factors refer to environmental conditions such as pH value, temperature, ionic strength, peptide concentration and solvent composition. These factors can be used to finely trigger, guide, or terminate the self-assembly process by adjusting the strength and properties of intermolecular interactions [57,58,59].

Therefore, SAPs demonstrate remarkable tunability and multifunctionality. Through precise molecular design and adaptable environmental regulation, supramolecular nanostructures can be rationally engineered to exhibit specific physicochemical properties and biological functions that meet desired requirements. This bottom-up construction strategy provides a powerful and flexible foundation for the fields of biomedical engineering, nanotechnology, and materials science [48,54,60].

### 3.1. The Driving Force of Peptide Self-Assembly

**Figure 2:**
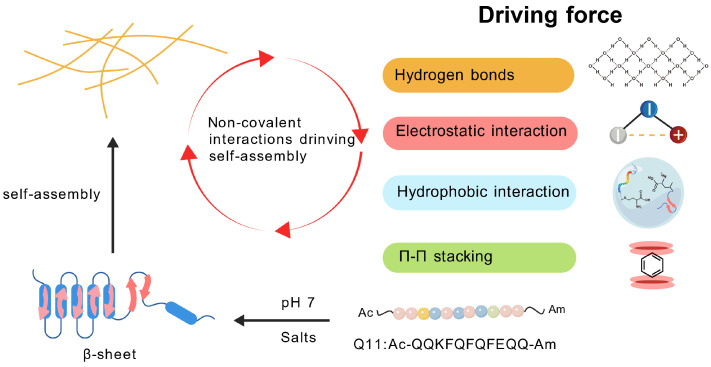
**Non-covalent force-driven peptide self-assembly.** At pH 7 and in the presence of salt ions, Q11 peptide adopts a β-sheet secondary structure and self-assembles into intertwined nanofibers through non-covalent interactions.

The self-assembly process of peptides follows a three-stage model of molecular recognition, reversible binding, and termination [61]. Although a single non-covalent interaction is relatively weak (<5 kcal/mol), the enthalpy change generated by the combined effect of many such interactions is sufficient to overcome the loss of entropy required for molecular ordering and formation of the final assembly [62]. Hydrogen bonding plays a key role in the formation of secondary structures in peptide chains, such as directional hydrogen bonding between adjacent peptide chains in beta folding or intramolecular hydrogen bonding in alpha helices. This provides the necessary directional constraints to induce the assembly of peptides into different nanostructures [63]. Hydrophobic interaction is the thermodynamic driving force for most self-assembly processes. It drives the aggregation of hydrophobic residues in order to minimize contact with water, and is significantly enhanced under salt screening conditions [15,64]. π-π stacking plays a crucial role in amino acids containing aromatic groups, such as phenylalanine and tyrosine. It contributes not only to the stabilization energy but also to orientational order during self-assembly. For example, the Fmoc-FF dipeptide forms antiparallel β-sheet nanostructures through interactions between the Fmoc group and benzene rings [65,66]. Electrostatic interactions regulate the assembly pathways through the attraction/rejection between charged residues, and their intensity is dynamically modulated by pH and ionic strength [67]. It is important to note that the aforementioned non-covalent interactions can act synergistically through chemical complementarity and structural compatibility, whereas kinetic factors modulate the assembly rate by affecting the frequency of molecular collisions, ultimately resulting in the formation of thermodynamically favored supramolecular assemblies [68] (Figure 2).

### 3.2. Environmental Factors Affecting Peptide Self-Assembly

#### 3.2.1. Temperature

Temperature significantly influences the kinetic pathway, nanostructure morphology, and thermal reversibility of peptide self-assembly by regulating the balance between molecular thermal motion and non-covalent interactions. The research found that the newly designed peptide AC-Pro-Ser-Phe-Cys-Phe-Lys-Phe-Glu-Pro-NH_2_ has the ability to self-assemble into fibers when the temperature is below 80 °C. However, when the temperature rises above 80 °C, the peptide solution predominantly forms irregular aggregates, and the fibrous morphology can no longer be observed [69]. In addition, research reported the effect of installing different lipids chains on the N-terminus of an octapeptide derived from the antiparallel β-interface of the diaminopimelate decarboxylase protein homotetramer. C_8_-HEFISTAH-NH_2_ exhibited pH and temperature stability with shear thinning properties suitable for biomedical applications [70]. Elastin-like polypeptides (ELPs), which are widely studied in temperature-responsive polypeptides, exist as monomers below the transition temperature but can transform into micelles upon heating. Moreover, their analogs have been developed for use as metal chelating agents [71,72]. These studies collectively demonstrate that temperature variations can exert diverse influences on the self-assembly behavior of peptides.

#### 3.2.2. pH

The pH can affect the electrostatic and hydrophobic interactions of peptides, and the amino acid side chains of peptides can exhibit different charge orientations under different pH conditions [73]. For instance, protonation of the central histidine in the alternating polarity peptide RFH (RFRHRHRFR) results in a 15% increase in solvent-accessible surface area (SASA), which leads to a significant reduction in DNA contacts and subsequent dissociation of the co-assembled complex. In contrast, the peripheral histidine in RHF (RHRFRFRHR) exhibits a more dispersed charge distribution, resulting in a weaker protonation effect and the maintenance of stable DNA binding capability [74]. The natural octapeptide PEP-1 forms a fractal structure dominated by β-sheets at pH 7.4. However, at pH 5.5, electrostatic repulsion is enhanced, causing it to transform into a random coil or helical structure, accompanied by the depolymerization of nanofibers [75]. A study designed a pH-responsive peptide (C16-VVAEEE), which can self-assemble into a hydrogel in an acidic environment [76]. In addition, Ye et al. identified three heptapeptides capable of self-assembling into nanofibers or nanoparticles at pH 7.5 and disassembling under acidic conditions (pH 6.5). Among these, P1 (LVEFRHY) exhibited a rapid response to acid stimulation and a pronounced morphological transition during the pH modulation process [77]. Therefore, by modulating the pH environment, SAPs with pH-dependent behavior can be rationally designed.

#### 3.2.3. Ionic Concentration

Ionic concentration remains a critical factor influencing the aggregation behavior of peptide molecules as well as the structural integrity and functional properties of proteins. Elevated ion concentration compresses the double-layer electrical barrier surrounding peptide chains, reduces intermolecular electrostatic repulsion—commonly referred to as the Debye–Hückel effect—and thereby markedly accelerates aggregation kinetics [78]. For example, in the absence of salt ions, the peptide MAX1 adopts a disordered conformation. However, upon the addition of a small quantity of salt, electrostatic interactions between the ions and charged amino acid residues induce the rapid formation of a β-hairpin structure, which further progresses into a β-sheet arrangement [64]. The above examples can be seen in Table 2. Moreover, the incorporation of metal ions has been shown to trigger conformational transitions in peptide self-assembly [79]. Ulijn et al. demonstrated that the addition of Cu^2+^ to the FFD-GHK co-assembly system induced a structural transformation of the peptide from nanofibers to hydrogels [80].

In addition to the above three main influencing factors, solvents and enzymes can also affect peptide self-assembly to a certain extent. Previous studies have reported that solvents can affect and alter the morphology of peptide self-assembly, and guide the chiral inversion of self-assembled peptide nanostructures [81,82]. Enzymes not only initiate the self-assembly process of peptides but also regulate the structural organization and morphological features of the resulting self-assembled peptide nanomaterials [83]. This indicates that researchers can design environmentally responsive self-assembling peptides by modulating the aforementioned factors, thereby enabling more effective functional performance.

### 3.3. The Unique Advantages of SAPs as Adjuvants

Non-biodegradable nanoparticles, like gold and silica, accumulate in the body over time, posing potential biotoxicity risks [84,85]. In contrast, self-assembling peptides are advantageous due to their simple synthesis process, reduced production complexity, and favorable safety profiles. Unlike virus-like particles (VLPs) or liposomes, self-assembling peptides avoid the need for complex structural components, offering a more biocompatible alternative with minimal risk of toxicity or long-term accumulation [86]. As a new-generation vaccine adjuvant platform, SAPs exhibit multiple unique advantages. Firstly, SAPs are composed of naturally occurring amino acids, and their metabolic byproducts exhibit excellent biocompatibility and safety. This intrinsic safety profile effectively circumvents the off-target effects commonly associated with synthetic adjuvants such as aluminum salts or Freund’s adjuvant. Through precise design of amino acid sequences, it is possible to modulate self-assembly behavior and nanostructure morphology, as well as enable environmental responsiveness, thereby achieving truly “tailor-made” functionalities. The three-dimensional network formed by them enables antigen encapsulation through covalent/physical mechanisms. Protecting the antigen from protease degradation while simultaneously enabling the co-delivery of antigen and adjuvant is critically important for eliciting a robust and efficient immune response [87]. SAPs activate innate immunity through distinct mechanisms. For instance, LL37/CpG co-assembled nanoparticles (L/C NPs) function as pathogen mimics, directly activating the TLR9 pathway via CpG and enhancing cellular uptake to amplify inflammation [88]. In contrast, an amphiphilic protein condensate strategy (PCD) induces organelle disruption by increasing mitochondrial membrane permeability, leading to mtDNA release and subsequent cGAS-STING pathway activation [89]. Furthermore, a dual-enzyme instructed self-assembly system (CPMC) triggers immunogenic cell death (ICD), coordinating the release of damage-associated molecular patterns to bridge innate and adaptive immunity [90].Additionally, many SAPs assemblies are capable of activating the immune system through the stimulation of TLRs and the NLRP3 inflammasome signaling pathways, eliminating the need for additional adjuvants and thereby circumventing the batch-to-batch variability associated with the chemical heterogeneity of conventional adjuvants [91]. In addition, the nanoscale size and surface charge characteristics of SAPs simulate pathogens, making them easily recognized and phagocytosed by antigen-presenting cells, which significantly enhances the efficiency of antigen presentation [30].

### 3.4. The Theoretical Basis of SAPs for Vaccine Research and Design

Peptide-based vaccines typically require three main components—antigens, adjuvants, and delivery carriers—to induce an effective adaptive immune response. When peptides serve as antigens, the conformation of the specific region that is recognized by the immune system—known as an epitope—plays a crucial role in eliciting humoral immunity. Peptide epitopes may interact with antibodies via α-helical, β-sheet/extended, or loop conformations. The precise conformation of the epitope within the antigen–antibody complex is essential for antibody function, as it enables specific recognition of the epitope within the structural framework of a folded globular antigen [92,93,94]. When designing vaccines, peptide self-assembly can be utilized to ensure the correct folding of antigenic epitopes. In vaccine applications where high antibody affinity and titer are critical, the incorporation of SAPs containing intrinsic CD4^+^ T cell epitopes—such as the Coil29 peptide (QARILEADAEILRAYARILEAHAEILRAD)—can effectively stimulate robust follicular helper T cell responses, thereby enhancing B cell activation and antibody production [32]. Similarly to the stimulation of CD4^+^ T cells, the peptide sequence is more capable of inducing cytotoxic T lymphocyte (CTL)-mediated cellular immunity than the conformation of the epitope itself. Therefore, shorter peptides can be effectively utilized to induce T-cell-mediated immune responses, as CD4^+^ T cells specifically recognize 12–16 amino acid-long peptides presented by MHC-II molecules on APCs, whereas CD8^+^ T cells target shorter peptides consisting of 8–10 amino acids that are bound to MHC-I [93,94]. In contrast to pathogen-based attenuated vaccines, peptide-based vaccines typically include adjuvants to potentiate the immune response against the antigen and to mimic the endogenous “danger signals” that occur during natural infection [95]. Furthermore, emerging evidence indicates that SAPs can function as adjuvants by facilitating the formation of antigen libraries, effectively directing vaccines to APCs, and thereby enhancing the activation of immune cells [96,97].

## 4. Application of SAPs in Vaccine Adjuvants

### 4.1. Adjuvant Properties of SAPs: Expanding and Enhancing Immune Responses Beyond Traditional Adjuvants

#### 4.1.1. Synergistic Activation of Balanced Th1/Th2 Immune Responses

The Ac-FFA-NH_2_ (HYG-1) nanofiber hydrogel enables sustained antigen release through the formation of a localized “antigen reservoir”, thereby efficiently recruiting and activating APCs. When loaded with ovalbumin (OVA), this system not only induces significantly higher total IgG titers compared to conventional adjuvants such as liposomes, MDP, and imiquimod, but more importantly, it promotes a balanced co-induction of both IgG1 (Th2-associated) and IgG2a (Th1-associated) antibodies. This dual induction underscores its superior ability to modulate immune balance, a feature seldom achieved by traditional adjuvants [98]. Similarly, Nano-B5 nanoparticles (20–50 nm), optimized for efficient self-assembly due to their size, exhibit enhanced lymph node targeting and significantly improve the uptake and activation of APCs. In immunization studies targeting Shigella flexneri O-polysaccharide (OPS), Nano-B5 induces markedly higher antibody titers than the aluminum adjuvant group, while simultaneously driving both Th1 and Th2 immune responses, thus overcoming the Th2-dominant immune bias typically associated with aluminum adjuvants [36]. Another notable example involves self-assembled nanoparticles displaying malaria Circumsporozoite Protein (CSP) epitopes: these nanoparticles present a highly ordered antigen array that directly activates B cells and provides effective T cell help independent of the TLR4 signaling pathway. These nanoparticles elicit protective antibodies with higher affinity and prolonged persistence compared to Montanide ISA-720, while completely avoiding the inflammatory side effects associated with traditional oil-based adjuvants [99].

#### 4.1.2. Efficient Activation of CTL Responses

The D-type tetrapeptide Nap-G^D^F^D^F^D^Y self-assembles into supramolecular nanofiber hydrogels that facilitate rapid antigen release within the acidic lysosomal environment and promote lysosomal escape of antigens, thereby enhancing MHC class I-mediated cross-presentation. Unlike aluminum-based adjuvants, which primarily stimulate humoral immunity, this platform not only amplifies anti-OVA IgG titers by 3.5-fold but, more importantly, elicits potent antigen-specific cytotoxic T lymphocyte (CTL) responses. In both B16-OVA and E.G7 tumor models, this results in marked tumor suppression, surpassing the therapeutic efficacy of MPLA [100]. The engineered peptide Ada-G^D^F^D^F^D^YG^D^KKK-NH_2_ (3DSNA), derived from a self-assembling motif, markedly enhances the uptake and cross-presentation of the model antigen ovalbumin (OVA), while simultaneously activating NF-κB signaling pathways. This dual mechanism leads to the robust induction of long-lasting antigen-specific memory and effector CD8^+^ T cell responses. Notably, when co-administered with OVA, 3DSNA outperforms conventional aluminum hydroxide adjuvants, resulting in a significant reduction in tumor incidence, suppression of tumor progression, and extended survival in melanoma-bearing murine models [101]. Similarly, Q11 nanofibers are efficiently internalized by dendritic cells following intranasal delivery and significantly enhance MHC-I-mediated cross-presentation, leading to robust antigen-specific CD8^+^ T cell responses and the generation of lung tissue-resident memory T cells in the absence of exogenous adjuvants, while avoiding the severe inflammatory reactions associated with LPS-based adjuvants [102]. In addition, α-helical Coil29 nanofibers exhibit inherent immunostimulatory properties that promote efficient antigen uptake by APCs and outperform the β-sheet-based Q11 platform in facilitating MHC-I-mediated antigen presentation. Notably, Coil29 induces interferon-γ (IFN-γ) secretion at levels comparable to those triggered by complete Freund’s adjuvant, underscoring its strong capacity to activate cellular immune responses [103].

#### 4.1.3. Efficient Induction of Mucosal Immune Responses

The uniform short nanorods (~100–200 nm) that self-assemble from M2e-KKI_10_ are well-suited for mucosal delivery. Intranasal immunization in the absence of exogenous adjuvants induces mucosal sIgA and systemic IgG levels comparable to those elicited by free M2e + Al, conferring robust protection against influenza A virus challenge. When combined with Montanide Gel, this formulation achieves a 100% survival rate in murine models [104]. The Coil29 platform, when administered via sublingual immunization, effectively reduces non-specific binding to mucin and enhances epithelial penetration through PAS (Sequence rich in proline, alanine and serine) modification, successfully stimulating both IgG and IgA responses at serum and mucosal sites (such as the vagina), thereby demonstrating its potential as a promising mucosal vaccine platform [33]. Similarly, PAS-Q11 nanofibers significantly improve mucosal permeability, facilitating their rapid transit through the gastrointestinal tract. When administered orally, PASylated nanofibers combined with mucosal adjuvants effectively trigger both local and systemic immune responses against peptide epitopes [105].

#### 4.1.4. Precise Immune Polarization and Epitope Synergy

The hydrogel formed by the co-assembly of FEFEFKFK with three tumor antigen epitopes exhibits a nanofiber structure that inherently possesses adjuvant activity, effectively promoting the maturation of DCs and facilitating antigen presentation. In the absence of exogenous adjuvants, it stimulates a broad-spectrum CD8^+^ T cell response, with its anti-tumor efficacy surpassing that of the combination of Montanide ISA-51 and CpG [106]. The co-assembly of CD8^+^ and CD4^+^T cell epitopes with the designed K/E peptide elicits robust anti-tumor T cell immunity, surpassing that induced by aluminum adjuvant, through selective activation of the MyD88–NF-κB signaling pathway without triggering detectable inflammatory responses [107]. Following intranasal immunization, Eα_52–68_ Q11 nanofibers are efficiently captured by pulmonary DCs, which subsequently migrate to draining lymph nodes and drive antigen-specific Th17-type immune responses, offering a promising strategy for the development of vaccines against respiratory infectious diseases [97].

### 4.2. The Role of SAPs’ Physical and Chemical Properties in Immune Regulation

#### 4.2.1. Size-Dependent Immune Activation

It has been reported that particles smaller than 200 nm are more efficiently internalized by APCs [108]. The high aspect ratio and dimensions of nanorods and nanofibers affect the uptake of antigens. Spherical particles or short rods measuring less than 50 nm are more easily internalized by cells. In contrast, elongated rods or fibers measuring between 50 and 200 nm are retained for longer within lymph nodes [109]. For example, uniform short nanorods (with a length of ~100–200 nm) that self-assembled from M2e-KKI_10_ induced high levels of mucosal sIgA and systemic IgG that were comparable to those observed in the experimental group that used Al as an adjuvant. This was achieved without the need for exogenous adjuvants. They also increased IgG2a levels and provided protection against the influenza virus [104]. Nano-B5 particles, ranging from 20 to 50 nm, demonstrate enhanced lymph node targeting, with accumulation in the draining lymph nodes reaching levels 14 times higher than that of free antigen within 6 h post-injection. Additionally, these particles significantly upregulate the expression of genes associated with antigen processing and presentation in DCs [36]. High-aspect-ratio nanostructures, exemplified by tyrosine-based peptide nanorods (YEF8, 20–30 nm in diameter and 500–1000 nm in length), can robustly activate DCs through a ‘frustrated phagocytosis’ mechanism, leading to substantial production of pro-inflammatory cytokines such as IL-1β and TNF-α. This activation drives M1-like macrophage polarization, as evidenced by elevated inducible nitric oxide synthase (iNOS) expression, thereby enhancing cell-mediated immune responses, including CD8^+^ T cell-mediated cytotoxicity—an effect desirable for cancer immunotherapy. In contrast, nanostructures with a low aspect ratio, such as EF8 peptide nanorods (3.8 nm in diameter and 20–40 nm in length), can penetrate macrophage membranes via a distinct mechanism, favoring M2-like polarization and promoting the secretion of anti-inflammatory cytokines, including IL-10 and TGF-β [110]. The immunogenicity of intact Q11 nanofibers is lost when they are sheared into shorter fibers with an average length of 82 nm. This emphasizes the critical importance of structural integrity in self-assembled architectures for inducing adaptive immune responses [102].

#### 4.2.2. Surface Charge as a Key Determinant of Intracellular Transport and Delivery Efficiency

Surface charge plays a pivotal role in governing the interactions between nanocarriers and cell membranes, as well as intracellular trafficking. D-type peptides undergo surface charge changes in the acidic environment of lysosomes, thereby facilitating the rapid release of antigens and lysosomal escape, which is a key mechanism for enhancing MHC-I cross-presentation [100]. The 4RDP (F5) peptide significantly enhanced its transmembrane and lysosomal escape capabilities through fluorination strategy, demonstrating a stronger cellular immune activation effect compared with aluminum adjuvant and CpG [87]. The PAS modification of Coil29 effectively enhanced its penetration and retention in mucosal tissues by reducing its ζ potential and regulating overall hydrophobicity, thereby improving mucosal immune efficacy [33].

#### 4.2.3. Molecular Conformation and the Diversity of Self-Assembly Driving Forces

Both α-helices and β-sheets have been shown to participate in the self-assembly of peptides in aqueous environments. Notably, β-sheets, due to their planar geometry and conformational flexibility, exhibit greater versatility in constructing a wide array of supramolecular nanostructures. In contrast, α-helices offer a distinct advantage in terms of superior environmental stability, outperforming β-sheets under various physiological conditions [111]. In comparison to the classic β-sheet conformation, the α-helix conformation (e.g., Coil29) has been shown to exhibit similarly potent built-in adjuvant activity. This effect is largely attributed to the T-cell epitopes embedded within the sequence and the potential activation of the STING pathway [103]. Furthermore, the functional controllability of SAPs can be achieved by modifying the tail domain of amphiphilic peptides [112]. Sequences rich in strong hydrophobic modules (such as KFE8, FEFEFKFK) mainly drive self-assembly through hydrophobic interactions. Among them, KFE8 enhances antigen presentation by activating the DAMPs pathway and the metabolic reprogramming of DCs, while avoiding excessive inflammation that may be caused by the TLR pathway [113]. As a DNA vaccine vector, KFE32 enhances MHC-II-mediated antigen presentation via the autophagy pathway when fused with GFP, thereby inducing a balanced Th1/Th2 immune response. Notably, it has been demonstrated to lack the cytotoxicity associated with pathological amyloid proteins [114]. Lipopeptide hydrogels (e.g., Myr-FF/FFY) integrate TLR2 agonist activity with sustained antigen release, and induce significantly higher levels of DCs maturation and pro-inflammatory cytokine secretion compared to aluminum adjuvants, achieving functional integration of adjuvant and delivery system properties [115].

### 4.3. SAPs: A Transition Toward Intelligent and Personalized Therapeutics

#### 4.3.1. Multifunctional Scaffolds for Co-Delivery and Immune Activation

RADA16 hydrogel, as a 3D scaffold, not only provides a suitable microenvironment for the loaded DCs and anti-PD-1 antibodies but also significantly promotes the recruitment and migration of endogenous DCs to lymph nodes, thereby amplifying antigen-specific T-cell immunity, with a superior effect compared to traditional immunization strategies [116]. Even more groundbreaking is the Fmoc-FF/PLL co-assembled hydrogel, which, without carrying any antigen or traditional adjuvant, directly activates T-cell-mediated anti-tumor immune responses. This is attributed to its helical nanofiber structure, which is recognized by the innate immune system as a “danger signal” [117]. Additionally, the PECT/EAASc self-assembled hydrogel system enables the co-localized delivery of chemotherapeutic agents and nano-vaccines at the tumor site, significantly reducing postoperative tumor recurrence and metastasis through spatiotemporal synergism [118]. Poly-L-leucine (PLLeu) is a poly-amino acid synthesized by linking L-leucine monomers through peptide bonds. Its self-assembly behavior is primarily driven by the balance between the hydrophobic side chains (isobutyl groups of leucine) and the hydrophilic backbone (peptide bonds), enabling the formation of diverse structures such as micelles, nanofibers, or nanovesicles. When copolymerized with poly-L-lysine (PLL), both of which are basic amino acids, PLLeu undergoes self-assembly into nanofibers or vesicles through electrostatic interactions or hydrogen bonding. For example, poly-lysine-poly-L-leucine (PLL-PLLeu) copolymers can form vesicles in aqueous environments that encapsulate Irinotecan, thus enhancing tumor-targeted drug delivery. This design strategy can be extended to encapsulate or conjugate vaccine antigens, offering protection against degradation and extending antigen exposure time [119].

#### 4.3.2. Integrated Antigen-Adjuvant Systems for Coordinated Delivery

**Figure 3:**
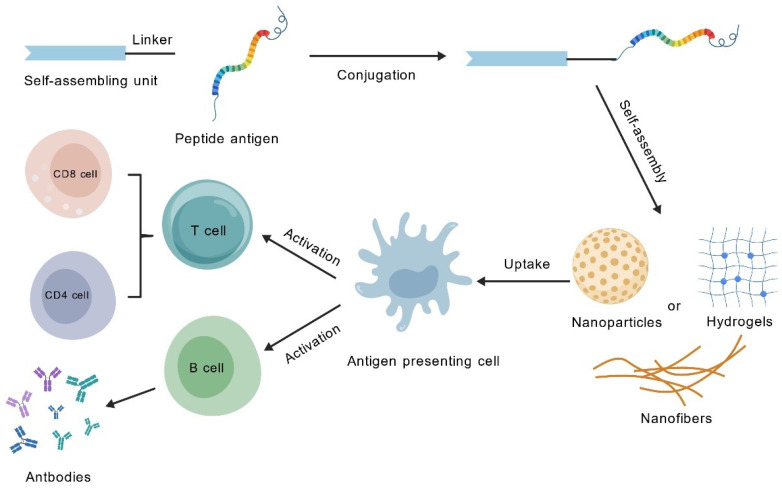
Mechanism diagram of self-assembled peptide basing vaccine.

Covalently conjugating antigen epitopes directly into the assembling units is a classic strategy in the design of SAP vaccines (Figure 3). For example, TBT-CpG NaVs covalently link the pan-epitope antigen with the CpG adjuvant via disulfide bonds and self-assemble into nanospheres. This design ensures the simultaneous uptake of both the antigen and the adjuvant by APCs, significantly enhancing DCs activation and antigen presentation, thereby generating robust cross-protection against related viruses [120]. Black et al. employed dipalmitoyl (diC16) lipid tails to facilitate the self-assembly of cylindrical micelles through their interaction with the CD8^+^ T-cell epitope SIINFEKL. This strategy was developed as an anti-cancer vaccine, effectively triggering cytotoxic T-cell responses and inhibiting tumor progression [121]. In a similar vein, the use of a platform based on the self-assembling peptide EAK16-II for the co-delivery of CD8^+^ T-cell epitopes and TLR7/8 agonists (such as R848 or R837) led to the elicitation of more robust, antigen-specific cytotoxic T lymphocyte (CTL) responses, both in vitro and in vivo [122]. Additionally, the OVA_323–339_-Q11 system demonstrates that simple covalent attachment of the antigen to the self-assembly domain can elicit an antibody response comparable to that induced by complete Freund’s adjuvant, through multivalent antigen display, while entirely avoiding the inflammatory side effects associated with the latter [19].

#### 4.3.3. Toward Clinically Relevant Smart Responsive and Personalized Platforms

**Figure 4:**
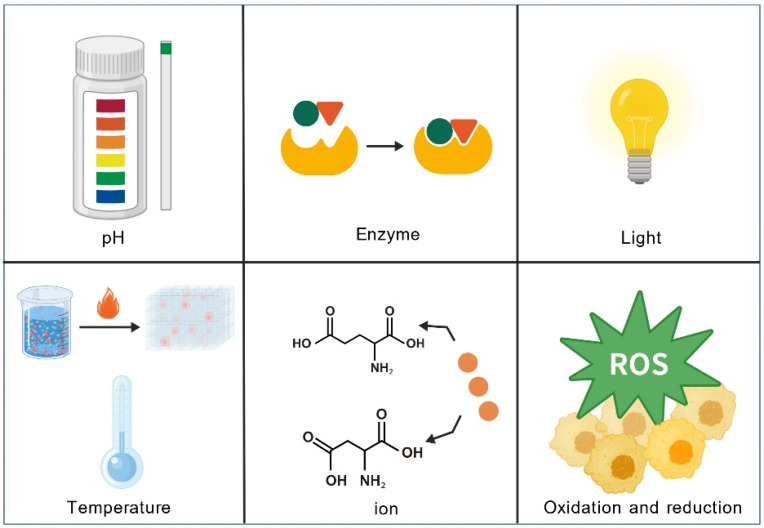
**Classification of stimulus-responsive self-assembled peptides.** Stimulus-responsive peptides can exhibit responsiveness to pH, enzymes, light, temperature, ions, and oxidative stress.

The PAC-SABI system undergoes in situ self-assembly into a nanofiber network on the surface of tumor cells upon activation by alkaline phosphatase. This assembly facilitates the simultaneous physical blockade of the dual “don’t eat me” signals mediated by CD47 and CD24, thereby synergizing with immune checkpoint inhibitors to enhance antitumor immunity [123]. In the realm of personalized cancer vaccines, the Fmoc-KCRGDK hydrogel encapsulates patient-derived inactivated tumor cells, immunomodulators, and photothermal agents, enabling controlled release triggered by near-infrared laser. This system synergistically promotes DCs maturation, reverses the immunosuppressive tumor microenvironment, and activates robust systemic antitumor immunity, thereby demonstrating a highly promising pathway toward truly personalized therapeutic strategies [124]. In the future, a wider variety of intelligent responsive systems will be applied to vaccine adjuvants, providing powerful tools for the development of next-generation precision immunotherapies (Figure 4 and Table 3).

## 5. Challenges and Future Outlook

SAP vaccine technology utilizes molecular self-organization to construct well-ordered nanostructures, enabling precise antigen delivery and robust immune activation. It has emerged as a pivotal strategy in the development of next-generation vaccines. SAPs as vaccine adjuvants demonstrate excellent safety profiles, showing no significant cytotoxicity, inflammatory responses, or systemic toxicity in in vivo experiments. For instance, when PAQ11 peptide nanofibers were administered intranasally to mice, local tissue inflammation assessments revealed no increased inflammatory cell infiltration, with key cytokine levels comparable to the negative control group, indicating good local tolerance [102]. This class of materials, characterized by low toxicity, high biocompatibility, and well-defined chemical structures, not only addresses the limitations of conventional adjuvants but also demonstrates unique self-adjuvant properties. The self-assembly process allows for the co-assembly of building blocks containing diverse epitopes into multi-antigen nanostructures, thus providing a promising pathway for the development of multivalent vaccines. Additionally, due to their inherent physicochemical properties, these systems can encapsulate both hydrophobic and hydrophilic therapeutics, facilitating stimulus-triggered drug release at the targeted disease site through the integration of responsive components within their design. However, SAPs still exhibits shortcomings, such as the relatively weak antibody response induced by the Q11-peptide-based malaria vaccine in mouse models, coupled with limited antibody persistence. These factors constrain its protective efficacy. Future research should focus on enhancing its immunostimulatory capacity through meticulous peptide sequence design, optimization of nanostructures, and the development of combination adjuvant strategies [125]. current understanding of the interactions between nanostructured vaccines and immune cell receptors remains limited. Critical gaps persist in research areas such as conjugate stability, APCs uptake efficiency, T-cell activation, and organ targeting specificity. Furthermore, SAPs, when employed as scaffold materials for in vivo drug delivery, are vulnerable to physiological conditions, which can undermine the stability of their self-assembled structures. Additionally, as most SAPs are synthesized via solid-phase peptide synthesis (SPPS), scaling up production faces several technical challenges. Although SAPs synthesis methods are simple and highly reproducible, their storage stability remains to be verified and optimized, necessitating greater regulatory oversight in clinical practice.

## 6. Conclusions

SAPs represent a promising and innovative class of next-generation vaccine adjuvant platforms, effectively integrating superior biomaterial characteristics with potent immunostimulatory capabilities, thereby overcoming many of the inherent limitations associated with conventional adjuvants. Through rational molecular design, SAPs hold the potential to precisely modulate both the nature and magnitude of immune responses to meet diverse disease-specific requirements, particularly exhibiting distinctive advantages in eliciting robust cellular immunity. Consequently, achieving comprehensive understanding and precise regulation of the self-assembly process is of paramount importance for future vaccine development. On the one hand, further investigation is required to develop nanostructures with precisely defined architectures and enhanced functional properties through rational peptide sequence design and optimized self-assembly conditions, aiming to improve vaccine immunogenicity and stability. On the other hand, comprehensive studies on the in vivo behavior of self-assembled peptide nanomaterials and their mechanistic interactions with the immune system are essential to establish a robust theoretical foundation for the rational design of next-generation vaccines. Furthermore, enhancing the clinical translation of SAPs requires addressing key challenges related to their in vivo stability and bioavailability, while improving safety and efficacy in clinical applications. This strategic advancement aims to facilitate the transition of SAP vaccines from bench to bedside, ultimately contributing to improved human health outcomes.

## Figures and Tables

**Table 1:** Classification of adjuvants.

Adjuvant Classification	Examples	Mode of Action	Approved Vaccines
Delivery system adjuvants	Aluminum adjuvants (aluminum hydroxide, aluminum phosphate)	Mainly induces Th2 type humoral immune response, but has weak cellular immune induction ability, which may cause local inflammatory response	Diphtheria, Tetanus, Pertussis (DTP) Vaccine and Hepatitis B Vaccine
	Emulsions (MF59, AS03)	Oil in water (o/w) or water in oil (w/o) type can enhance humoral and certain cellular immunity	AS03 is used for influenza vaccines and H5N1 avian influenza vaccines
	Microparticle/Nanoparticle Delivery Systems (VLPs, Lipid Nanoparticles, Liposomes)	Simulating pathogens, easily ingested by APCs, with both delivery and immune stimulation functions	COVID-19 Vaccine, Shingles Vaccine
Immunostimulants	TLR Agonists (MPLA, CpG ODN, Flagellin)	Specific activation of PRRs, induction of chemokine and inflammatory cytokine production, T cell immune bias	Combined with aluminum for hepatitis B vaccine and COVID-19 vaccine (CpG 1018)
	Cytokines (IL-2, IL-12)	Regulate the function, activation, and differentiation direction of immune cells	_
	Chitosan	Activation of cGAS STING and NLRP3 inflammasome pathways can enhance Th1 type immune response	_
Compound adjuvants	AS01	Improve antibody titers and enhance Th1 type immune responses	Shingles Vaccine, Malaria Vaccine
	AS02		_
	AS04	Activate TLR4, promote the maturation of APCs, and improve humoral and cellular immunity	Human papilloma (HPV) vaccine, hepatitis B vaccine
Novel Biomaterial Adjuvants	Self-Assembling Peptides, Polymeric Nanoparticles	Degradable, structurally and functionally designable, capable of integrating antigen delivery and immune regulatory functions	_

**Table 2:** SAPs mentioned in Section 3.2.

Peptide Sequence	Nanostructure	Features	Ref.
PSFCFKFEP	Nanofiber hydrogel	Temperature < 80 °C, nanofiber; Temperature > 80 °C, Irregular aggregates	[69]
C_8_-HEFISTAH-NH_2_	Hydrogel	Stable in pH 4–8 and temperature 25–50 °C range	[70]
Elastin-like polypeptides (ELPs) G-(QYPSDGRG)n-(XGVPG)m-Y	Spherical micelle, Cylindrical micelles	temperature response	[71]
(FPGVG)n	Micron-sized spherical aggregates	temperature response	[72]
RFH: NH_3_^+^-RFRHRHRFR-COO^−^	Nanostructure	pH response	[74]
Histidine repeat sequence (12×His)	Spherical nanoparticles	pH response	[75]
C16-VVAEEE	Nanofiber hydrogel or solution	pH ≤ 6.8, nanofiber hydrogel; pH ≥ 7.0, solution	[76]
P1 (LVEFRHY)	Nanofiber	pH 7.5, nanofibers; pH 6.5, nanoparticles	[77]
FFD (Phe-Phe-Asp) GHK (Gly-His-Lys)	Hydrogel	At pH 7.4, GHK formed amorphous aggregates, while FFD assembled into nanofibers. When Cu^2+^ (30 mM) was added, the FFD/GHK mixture rapidly formed a three-dimensional nanofiber network, and transformed into a hydrogel	[80]
VKVKVKVK-VPPPT-KVKVKVKV	Hydrogel	At pH 7.4, adding salts (e.g., NaCl or KF) shielded the electrostatic repulsion of lysine (K) residues, thereby inducing protein folding	[64]

**Table 3:** Specific information on the application of SAPs in vaccine adjuvants.

Peptide Name and Sequence	Self-Assembly Type	Immunization Strategy/Application	Key Results	Reference
Q11 (Ac-QQKFQFQFEQQ-Am)	β-sheet nanofibers	Insert hydrophilic SGSG linker between OVA and Q11 domains to form O-Q11; Subcutaneous injection	Elicited high IgG titers similar to CFA; dependent on self-assembly; no significant T cell help involved.	[19]
		Connecting pEα peptide antigen through SGSG to form E_α52–58_-Q11, Intranasal immunization	Induced lung dendritic cell activation and migration to lymph nodes; primed TH17 responses independently in lung and lymph nodes.	[97]
		Oral immunization with PASylation modifications for mucosal delivery against peptide (OVA_323–339_) and small molecule (phosphorylcholine) epitopes.	Enabled oral immunization by resisting protease degradation and enhancing muco-penetration; induced systemic and local immune responses without inflammation; effective in DSS colitis models.	[105]
	Nanofibers	The Q11 domain and acidic polymerase (PA_224–233_, SSLENFRAYV) are linked by SGSG to form the PAQ11 peptide; Intranasal delivery; influenza vaccine.	Elicited resident CD8+ T cells in lung, non-inflammatory, provided protection against influenza challenge.	[102]
Coil29 (QARILEADAEILR-AYARILEAHAEILRAQ)	α-helical nanofibers	Sublingual immunization with epitopes (e.g., OVA, 2C7, FP)	Raised robust immune responses; PASylation required for hydrophobic epitopes to reduce mucin complexation and enhance epithelial penetration.	[33]
		Subcutaneous immunization in mice with epitope-bearing nanofibers (e.g., PEPvIII, PADRE, SIINFEKL) for cancer and model antigens.	Induced robust antibody, CD4^+^ T-cell, and CD8^+^ T-cell responses without supplemental adjuvants; antibody titers higher than CFA-adjuvanted groups; promoted epitope uptake by APCs.	[103]
Nano-B5 platform (e.g., CTB-Tri, LTB-Tri, StxB-Tri)	Nanoparticle	Based on AB5 toxins and trimer-forming peptides; used for prophylactic and therapeutic vaccines against infections and tumors	Induced strong humoral and cellular immune responses in mice and monkeys; excellent lymph node targeting and safety.	[36]
4RDP (F5) (containing di-pentafluorophenylalanine (F5) and tetra-arginine (4R))	Nanoparticle	Fluorinated supramolecular self-assembly, as adjuvant for cancer therapy (e.g., with OVA antigen)	Enhanced antigen uptake, lysosomal escape, and cross-presentation; elicited TH1-biased cellular immunity; combined with anti-PD-L1 for tumor inhibition.	[87]
Ac-FFA-NH_2_	Hydrogel	Subcutaneous delivery with OVA antigen; vaccine adjuvant	Induced high IgG titers, robust humoral and cellular immune responses; composite with liposomes showed sustained antigen release.	[98]
P4c-Mal (100 amino acid monomeric linear peptide)	Nanoparticles	Subcutaneous injection without adjuvant; malaria vaccine	Long-lasting protection against Plasmodium berghei, high antibody titers, CD4^+^ T cell-dependent response.	[99]
Nap-GFFY	Hydrogel	Subcutaneous immunization; vaccine adjuvant for cancer and infectious diseases	Stimulated strong CD8^+^ T cell responses, enhanced antigen uptake by dendritic cells, non-inflammatory.	[100]
3DSNA (Ada-G^D^F^D^F^D^YG^D^K^D^K^D^K-NH_2_)	Nanofibers (pH-triggered self-assembly)	Subcutaneous injection; cancer immunotherapy adjuvant	Activated NF-κB, enhanced antigen presentation, induced CD8+ T cell responses, inhibited tumor growth.	[101]
KKI_10_ (KKGSGSSNNFGAILSS)	β-sheet nanorods	Highly conserved epitopes connecting extracellular domains of matrix protein 2 (M2e); Subcutaneous and intranasal immunization.	Elicited M2e-specific IgG responses; provided complete protection against influenza A virus (H1N1); self-adjuvanting with efficient APC uptake and TLR-2 activation.	[104]
F peptide (FEFEFKFK)	Nanofibrous hydrogel	F peptide co-assembled with gp100_209–217_, Tyr_369–377_, MART_-126–35_, Subcutaneous immunization in mice for cancer immunotherapy (B16 melanoma).	Enhanced DC maturation and antigen presentation; elicited broad-spectrum CD8+ T-cell responses; inhibited tumor growth without additional adjuvants.	[106]
K-peptide (KWKAKAKAKWK)and E-peptide (EWEAEAEAEWE)	Nanofibrous hydrogel	OVA_323–336_ is covalently linked to the C-terminus of K or E peptides via GGG linkers, forming epitope conjugated peptides (ECPs); Subcutaneous immunization in mice for cancer immunotherapy (E.G7-OVA lymphoma).	Activated MyD88-dependent NF-κB pathway in DCs without inflammation; enhanced T-cell immunity and tumor inhibition; self-adjuvanting.	[107]
EF8 (EFEFKFEFK)	β-sheet nanofibers	In vitro treatment of THP-1 derived macrophages or PBMC-derived macrophages at 2 mM or 20 mM	Induced M2c polarization (anti-inflammatory response)	[110]
YEF8 (YEFEFKFEFK)	β-sheet nanofibers	In vitro treatment of THP-1 derived macrophages or PBMC-derived macrophages at 2 mM or 20 mM Intratracheal booster in BCG-primed mice	Induced M1 polarization (pro-inflammatory response)	
EF8Y (EFEFKFEFKY)			No strong inflammatory response, tends towards M2a-like state	
YEF8Y (YEFEFKFEFKY)			No significant inflammatory response	
EYF8 (EYEFKFEFK)			No significant inflammatory response, tends towards M2a-like state	
KFE8-Ag85B (KFE8: FKFEFKFE conjugated to Ag85B_240–254_via cleavable linker)			Increased frequency of Ag85B-specific CD4^+^T cells, including tissue-resident memory cells; no improved protection against Mtb challenge	
KFE32-GFP (KFE32: 4 repeats of KFE8 with linkers, fused to GFP)	Nanofibers (in vivo likely)	DNA vaccine, intramuscular injection in mice	Elicited anti-GFP antibodies and CD8^+^ T cell responses; balanced Th1/Th2 response	[114]
Myr-FF (Myristic acid-Phe-Phe)	Lipopeptide hydrogel	Used as an adjuvant for delivering GPC-3 peptide antigen in cancer vaccines	Acted as a TLR2 agonist, upregulates costimulatory molecules (CD80, CD83, CD86) on DCs, induced cytokine secretion (e.g., IL-6, TNF-α), and promoted leukocyte infiltration in lymph nodes without toxicity.	[115]
Myr-FFY (Myristic acid-Phe-Phe-Tyr)	Lipopeptide hydrogel	Same as above; adjuvant for GPC-3 peptide delivery	Showed higher TLR2 activation compared to Myr-FF, enhanced DC maturation, and sustained release of antigen with robust immune response.	
RADA16 (Ac-RADARADARADARADA-NH2)	Nanofibrous hydrogel	Simple physical mixture of peptide nanofiber hydrogel, anti PD-1 antibody, dendritic cells and tumor antigen is injected subcutaneously	Enhanced DC maturation and antigen presentation, recruited host DCs, promotes T-cell proliferation and cytokine secretion (e.g., IFN-γ), and suppressed tumor growth in prophylactic and therapeutic models.	[116]
Fmoc-FF (Fmoc-Phe-Phe)	Hydrogel	Injectable peptide hydrogels with adjustable mechanical and rheological properties were obtained by electrostatic coupling and co assembly with positively charged poly lysine (PLL)	Induced T cell activation (increasing CD4^+^ and CD8^+^T cells), inhibited tumor growth, and exhibited biocompatibility and biodegradability in vivo without the addition of antigens, immune regulatory factors, and adjuvants.	[117]
TBT (KYVKQNTLKLAT-GGVDRGWGNGCGLFGKG-LL-LEYIPEITLPVIAALSIAES)	Nanoparticles	Combining TBT with adjuvant CpG to form nanovaccine; Subcutaneous immunization in mice	Enhanced antigen-specific IgG, increased IFN-γ and IL-4 expression, protection against DENV and ZIKV	[120]
J8 (QAEDKVKQSREAKKQVEKALKQLEDKVQK)	Cylindrical micelles	J8 peptide covalently couples with dipalmitoylglutamic acid (diC_16_) to form J8-diC_16_, Subcutaneous vaccination in mice	Induced strong IgG1 antibody response comparable to conventional adjuvants	[121]
EAK16-II: AEAEAKAKAEAEAKAK	Nanofibers	The coupling of the EAK16-II peptide with the HIV-1-specific CTL epitope SLYNTVATL produced SL9-EAK16-II. This was then co-assembled with the TLR7/8 agonist R848 to create a tripartite formulation, In vitro DC stimulation and mouse immunization	Promoted DC maturation and specific CTL response	[122]
PAC-SABI (FFVLKAWSATWSNpYWRH)	Nanofiber network	In vitro and in vivo tumor models	Simultaneous blocking of CD47/CD24 signals enhanced macrophage phagocytosis	[123]

## Data Availability

No new data were created or analyzed in this study.
